# Interactional competencies in medical student admission at the Medical Faculty Heidelberg

**DOI:** 10.3205/zma001768

**Published:** 2025-09-15

**Authors:** Clara Schütte, Stefan Teichert, Jobst-Hendrik Schultz, Tim Wittenberg, Sabine C. Herpertz

**Affiliations:** 1 Heidelberg University, Medical Faculty heiTEST, Heidelberg, Germany

**Keywords:** interactional skills, multiple mini-interview, doctor-patient relationship, allocation of study places, additional aptitude quota

## Abstract

Since 2023, the multiple-mini-interview-based selection procedure *Interactional Competencies Medicine* (IC-MD) has been used in the Additional Aptitude Quota 1 (ZEQ-1) in Heidelberg. The IC-MD enables the identification of applicants who get access to medical studies through outstanding interactional skills. These skills are important for both studying as well as practicing as a doctor, as they are among the most relevant characteristics of what makes a good medical doctor. The IC-MD assesses these skills based on the concept of emotional availability and is evaluated by trained raters using a detailed manual. Data from a pilot study from 2019-2021 has now been used to analyze the stability of the measured interactional competencies (*r*=0.40) with follow-up assessments based on the IC-MD manual as part of the OSCE (*Objective Structured Clinical Evaluation*) examinations. Results show that interactional competencies are relatively stable over time: Students who demonstrate excellent interactional competencies at the beginning of their studies maintain those throughout their studies.

## Abstract (de)

In Heidelberg wird seit dem Jahr 2023 in der Zusätzlichen Eignungsquote (ZEQ-1) das Multiple-Mini-Interview basierte Verfahren *Interaktionelle Kompetenzen Medizin* (IKM) eingesetzt. Hier ermöglicht der Einsatz des IKM, Bewerbende zu identifizieren, die einen Zugang zum Studium durch besondere interaktionelle Kompetenzen erhalten. Diese Kompetenzen sind sowohl für das Studium als auch den Beruf angehender Mediziner*innen von Bedeutung, denn sie zählen zu den relevantesten Eigenschaften guter Ärzt:innen. Das IKM erfasst diese Fähigkeiten basierend auf dem Konzept der emotionalen Verfügbarkeit und wird von geschulten Rater:innen anhand eines detaillierten Manuals bewertet. Durch Daten einer Pilotierungsstudie von 2019-2021 wird nun mit erneuten Bewertungen anhand des IKM-Manuals im Rahmen der OSCE-Prüfungen die Stabilität der gemessenen interaktionellen Kompetenzen analysiert (*r*=0.40) welche zeigt, dass interaktionelle Kompetenzen über die Zeit relativ stabil sind: Studierende, die zu Beginn des Studiums hervorragende interaktionelle Kompetenzen vorweisen, zeigen dies auch im Verlauf des Studiums.

## Introduction

Every year, Heidelberg University receives around 20,000 applications for medical studies. The application process is centrally managed via different quotas: In the quota *University Selection Procedure* (AdH), *high school grade point average* (GPA) and the* Test for Medical Studies* (TMS) are weighted almost equally; in the *Abitur best quota*, the selection is based solely on GPA, without consideration of the TMS. In the *Additional Aptitude Quota 2* (ZEQ-2), selection is mainly based on the TMS, without consideration of GPA. Around 700 people additionally apply to participate in the procedure Interactional Competencies Medicine (IC-MD). Since 2023, the IC-MD has been used as the only selection criterion in ZEQ-1, with a pre-selection based on the TMS result. Accordingly, due to the high number of applicants, they are invited to participate based on their TMS results. Five percent of study places (15 places) are currently allocated via this oral procedure. An analysis of the allocation of study places in other main quotas, such as the Abitur best quota and the AdH or ZEQ-2, shows that several applicants who were successful in the IC-MD would not have received a place to study medicine in other quotas. Applicants with a GPA of up to 3.0 could thus be admitted to the program (see table 1 (Tab. 1)). The IC-MD shows no correlation with the results of the TMS and GPA [1]. Instead, the IC-MD enables the identification of applicants who have study- and career-relevant competencies due to their outstanding interactional skills and thus gain access to medical education. This shows the importance of the IC-MD as a valuable supplementary tool in the allocation of medical study places, as it complements the overall student selection strategy. While other quotas primarily consider applicants with a GPA above-average and excellent TMS results, thus focusing on cognitive abilities in the student selection process, the IC-MD supports applicants with excellent interactional skills, in addition to their cognitive abilities which are ensured through the TMS pre-selection.

## Project description

Research in the field of interactional video analysis characterize interactional competencies as contextual skills used in social interactions to engage in certain social practices [2], [3], [4]. These skills are not only crucial for academic success, but also for later medical practice, as interactional skills are among the most important characteristics of good doctors. Interactional skills of physicians have a significant influence on patient satisfaction, treatment adherence, self-reported health-related quality of life as well as patient symptoms and behaviour [4], [5], [6]. In addition, they are also seen as relevant from other stakeholders such as medical staff, medical students, and the general population [2], [3]. Internationally, the Multiple Mini Interview (MMI) has been established for evaluating these competencies [7] and is becoming increasingly important in the selection of medical students. At Heidelberg University, the MMI-based selection procedure IC-MD is assessing applicants in a series of short interviews with actors as patients. Clinical interactions are simulated, whereby interactional skills are assessed and not medical expertise. Applicants are only allowed to participate once. While earlier MMIs usually relied on expert consensus instead of being developed according to a clearly defined theoretical or even empirically founded construct, the IC-MD is based on the empirically validated concept of Emotional Availability (EA [8]). EA stems from attachment theory and has already been extended to social communication in a clinical context [9], [10]. It comprises communicative skills that enable the development of a stable relationship characterized by trust, is empirically based, and can be easily assessed in realistic contexts. Interactions are video-recorded and subsequently evaluated in a standardized manner by trained raters with a medical or psychological background. This assessment is carried out according to a manual on emotional availability comprising the four main scales of sensitivity, structuring, non-intrusiveness, and non-hostility. Each main scale consists of five subscales. All scales jointly result in the IC-MD score. The applicants with the best interactional skills, in addition to their excellent cognitive abilities already confirmed based on the TMS result, are then selected. 

## Results

In a longitudinal study, we re-assessed the interactional skills tested with the IC-MD to examine their development over the course of medical studies. This is important for the question whether the IC-MD results as part of the application process allow conclusions about relatively stable competencies reflected in academic performance. 123 participants from the IC-MD pilot study (2019-2021) were re-evaluated in their third year of study, after they had already received practical communication training. In their OSCE examinations on the subject of internal medicine, students were assessed by certified IC-MD raters in four communicative stations on the four main scales of the IC-MD using the same rating manual. Results showed a significant correlation between the IC-MD results before the start of the study and the interactional skills measured in the OSCE examinations, both overall (*r*=.40**) and for each of the subscales *sensitivity* (*r*=.41**), *structuring* (*r*=.42**), *non-intrusiveness* (*r*=.28**) and *non-hostility* (*r*=.26**). 

## Discussion and conclusion

Our findings indicate a high stability of interactional competencies measured by the IC-MD across contexts. They suggest that students with good socio-communicative skills at the beginning of their studies keep demonstrating these competencies throughout their studies. Conversely, students who are less able to present good socio-communicative skills at the beginning continue to have deficits, despite courses aimed at practicing communicative skills. This result supports the usefulness of the selection procedure in Heidelberg in the ZEQ-1 quota in admitting applicants who possess strong socio-communicative skills, a profile often desired for the medical profession. Additional follow-up measurements at the end of the program and afterwards are of interest to further corroborate this finding.

## Authors’ ORCIDs

- Clara Schütte: [0009-0005-6244-8219]
- Jobst-Hendrik Schultz: [0000-0001-9433-3970]
- Tim Wittenberg: [0000-0002-9295-4121]
- Sabine C. Herpertz: [0000-0001-9676-1928]

## Acknowledgement

We acknowledge financial support by Heidelberg University for the publication fee.

## Competing interests

The authors declare that they have no competing interests. 

## Figures and Tables

**Table 1:**
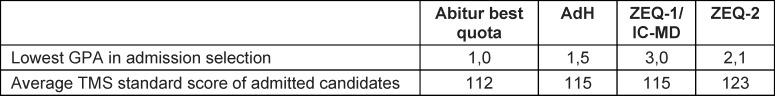
GPA and TMS results in the various selection quotas
